# Utilizing large language models for detecting hospital-acquired conditions: an empirical study on pulmonary embolism

**DOI:** 10.1093/jamia/ocaf048

**Published:** 2025-03-19

**Authors:** Cheligeer Cheligeer, Danielle A Southern, Jun Yan, Guosong Wu, Jie Pan, Seungwon Lee, Elliot A Martin, Hamed Jafarpour, Cathy A Eastwood, Yong Zeng, Hude Quan

**Affiliations:** Centre for Health Informatics, Cumming School of Medicine, University of Calgary, Calgary T2N 4N1, Canada; Provincial Research Data Services, Alberta Health Services, Calgary T2N 4N1, Canada; Centre for Health Informatics, Cumming School of Medicine, University of Calgary, Calgary T2N 4N1, Canada; Concordia Institute for Information Systems Engineering, Concordia University, Montreal H3G 2W1, Canada; Centre for Health Informatics, Cumming School of Medicine, University of Calgary, Calgary T2N 4N1, Canada; Department of Community Health Sciences, Cumming School of Medicine, University of Calgary, Calgary T2N 4N1, Canada; Centre for Health Informatics, Cumming School of Medicine, University of Calgary, Calgary T2N 4N1, Canada; Department of Community Health Sciences, Cumming School of Medicine, University of Calgary, Calgary T2N 4N1, Canada; Centre for Health Informatics, Cumming School of Medicine, University of Calgary, Calgary T2N 4N1, Canada; Provincial Research Data Services, Alberta Health Services, Calgary T2N 4N1, Canada; Centre for Health Informatics, Cumming School of Medicine, University of Calgary, Calgary T2N 4N1, Canada; Provincial Research Data Services, Alberta Health Services, Calgary T2N 4N1, Canada; Concordia Institute for Information Systems Engineering, Concordia University, Montreal H3G 2W1, Canada; Centre for Health Informatics, Cumming School of Medicine, University of Calgary, Calgary T2N 4N1, Canada; Department of Community Health Sciences, Cumming School of Medicine, University of Calgary, Calgary T2N 4N1, Canada; Concordia Institute for Information Systems Engineering, Concordia University, Montreal H3G 2W1, Canada; Centre for Health Informatics, Cumming School of Medicine, University of Calgary, Calgary T2N 4N1, Canada; Department of Community Health Sciences, Cumming School of Medicine, University of Calgary, Calgary T2N 4N1, Canada; Libin Cardiovascular Institute, University of Calgary, Calgary T2N 4N1, Canada

**Keywords:** adverse event detection, large language models, clinical text mining, pulmonary embolism

## Abstract

**Objectives:**

Adverse event detection from Electronic Medical Records (EMRs) is challenging due to the low incidence of the event, variability in clinical documentation, and the complexity of data formats. Pulmonary embolism as an adverse event (PEAE) is particularly difficult to identify using existing approaches. This study aims to develop and evaluate a Large Language Model (LLM)-based framework for detecting PEAE from unstructured narrative data in EMRs.

**Materials and Methods:**

We conducted a chart review of adult patients (aged 18-100) admitted to tertiary-care hospitals in Calgary, Alberta, Canada, between 2017-2022. We developed an LLM-based detection framework consisting of three modules: evidence extraction (implementing both keyword-based and semantic similarity-based filtering methods), discharge information extraction (focusing on six key clinical sections), and PEAE detection. Four open-source LLMs (Llama3, Mistral-7B, Gemma, and Phi-3) were evaluated using positive predictive value, sensitivity, specificity, and F1-score. Model performance for population-level surveillance was assessed at yearly, quarterly, and monthly granularities.

**Results:**

The chart review included 10 066 patients, with 40 cases of PEAE identified (0.4% prevalence). All four LLMs demonstrated high sensitivity (87.5-100%) and specificity (94.9-98.9%) across different experimental conditions. Gemma achieved the highest F1-score (28.11%) using keyword-based retrieval with discharge summary inclusion, along with 98.4% specificity, 87.5% sensitivity, and 99.95% negative predictive value. Keyword-based filtering reduced the median chunks per patient from 789 to 310, while semantic filtering further reduced this to 9 chunks. Including discharge summaries improved performance metrics across most models. For population-level surveillance, all models showed strong correlation with actual PEAE trends at yearly granularity (r=0.92-0.99), with Llama3 achieving the highest correlation (0.988).

**Discussion:**

The results of our method for PEAE detection using EMR notes demonstrate high sensitivity and specificity across all four tested LLMs, indicating strong performance in distinguishing PEAE from non-PEAE cases. However, the low incidence rate of PEAE contributed to a lower PPV. The keyword-based chunking approach consistently outperformed semantic similarity-based methods, achieving higher F1 scores and PPV, underscoring the importance of domain knowledge in text segmentation. Including discharge summaries further enhanced performance metrics. Our population-based analysis revealed better performance for yearly trends compared to monthly granularity, suggesting the framework's utility for long-term surveillance despite dataset imbalance. Error analysis identified contextual misinterpretation, terminology confusion, and preprocessing limitations as key challenges for future improvement.

**Conclusions:**

Our proposed method demonstrates that LLMs can effectively detect PEAE from narrative EMRs with high sensitivity and specificity. While these models serve as effective screening tools to exclude non-PEAE cases, their lower PPV indicates they cannot be relied upon solely for definitive PEAE identification. Further chart review remains necessary for confirmation. Future work should focus on improving contextual understanding, medical terminology interpretation, and exploring advanced prompting techniques to enhance precision in adverse event detection from EMRs.

## Introduction

Case identification is crucial in clinical care and research for identifying specific patients from electronic medical records (EMRs) based on predefined clinical or diagnostic criteria.^1^ A key focus is identifying adverse events, which are undesirable experiences associated with medical products or services, that can range from acute kidney injury^2^ to patient falls^3^ and diagnostic errors.^4^ Accurate and timely detection of these adverse events is essential for patient safety monitoring, quality improvement, and regulatory compliance.

Traditionally, case identification has relied on rule-based methods using structured data, such as the International Classification of Diseases (ICD) codes from administrative health data and keywords from clinical notes.^5–8^ While systematic, these approaches are often rigid and fail to capture the complexity of clinical documentation.^6^^,^^9^^,^^10^ More recently, researchers have explored machine learning-based approaches using supervised learning algorithms trained on annotated datasets.^3^^,^^11–13^ However, these models require large volumes of labeled data, which are resource-intensive and particularly difficult to obtain for hospital adverse events.

Pulmonary embolism as an adverse event (PEAE) presents a notable challenge in detection due to its complex causality and timing. Pulmonary embolism is a leading preventable cause of hospital death. It occurs when a blood clot obstructs a lung artery, with an annual incidence of 1-2 per 1000 patients in Canada.^14^^,^^15^ Identifying PEAE requires distinguishing hospital-acquired cases from community-acquired ones, establishing causality with medical procedures, and analyzing unstructured clinical documentation.^16^ In this study, we focus exclusively on differentiating PEAE from non-PEAE cases, where non-PEAE encompasses both patients without evidence of PE and those with PE not linked to an adverse event.

Given the complexities of the PEAE detection task, we propose a large language model (LLM)-based framework that leverages natural language processing to analyze unstructured clinical narratives. Pulmonary embolism as an adverse event serves as a representative case due to its reliance on temporal reasoning, causality assessment, and detailed documentation analysis. Beyond PEAE, our framework can be adapted for detecting other adverse events with similar challenges, such as hospital-acquired infections, medication-related complications, and post-procedural adverse events, by modifying the prompts and settings. This adaptable approach enables broader application in adverse event detection across diverse clinical scenarios.

We evaluate several pre-trained LLMs, including Llama3,^17^ Mistral 7B,^18^ Gemma,^19^ and Phi-3,^20^ to extract essential information from clinical notes, treatment records, diagnostic tests, and event timelines. By systematically evaluating these models across diverse configurations, we aim to identify the most effective approach for PEAE detection in our EMR text database. This study seeks to develop a robust, scalable solution that enhances detection capabilities while reducing reliance on large, manually labeled datasets, improving efficiency and adaptability in real-world clinical applications.

## Methods

### Study design and cohort

The proposed LLM framework was developed and tested within a secure computing environment, adhering to local health legislation and guidelines set by health authorities. It focused on adult patients aged 18 to 100 years who were admitted to hospitals in Calgary, Alberta, Canada. Patients without documented discharge summaries were excluded. A chart review cohort of patients randomly selected from Calgary’s tertiary-care hospitals between 2017 and 2022 was created. For patients with multiple hospitalizations, only the most recent admission was retained. Details have been reported elsewhere.^21^

The chart review was conducted by six registered nurses, following a predefined protocol for reviewing adverse events, as defined by our team.^22^ This included 18 categories of adverse events such as falls, sepsis, surgical site infections, and PE. The guidelines were established before beginning the chart review process. The chart reviewers were tasked with extracting basic patient demographic information, social determinants of health, comorbidities, and adverse events.

Understanding the association between adverse events and healthcare-associated factors is essential for determining whether conditions like PE should be classified as adverse events.^16^^,^^23^^,^^24^ Temporal inference focuses on establishing whether PEAE occurred before or during the hospital stay.^24–26^ The chart reviewers designated patients with confirmed PEAE as positive cases in their comprehensive review. All other scenarios, including PE present on admission, PE resulting from non-hospital-related events, and cases where no PE was found, were classified as negative cases.

### Framework rationale

Detecting adverse events, particularly PEAE, requires a two-step analytical approach. The first step determines whether a PE was diagnosed during the hospitalization and establishes its temporal relationship to admission—specifically, whether it was present on admission or developed during the hospital stay. For cases where PE developed after admission, the second step evaluates whether it meets the criteria for a hospital-acquired adverse event by considering both timing and its relationship to medical care or procedures. This systematic evaluation process mirrors the approach used by chart reviewers in their manual assessment, distinguishing community-acquired PE from those that developed as potential adverse events during hospitalization.

### Framework architecture

**Figure 1.**
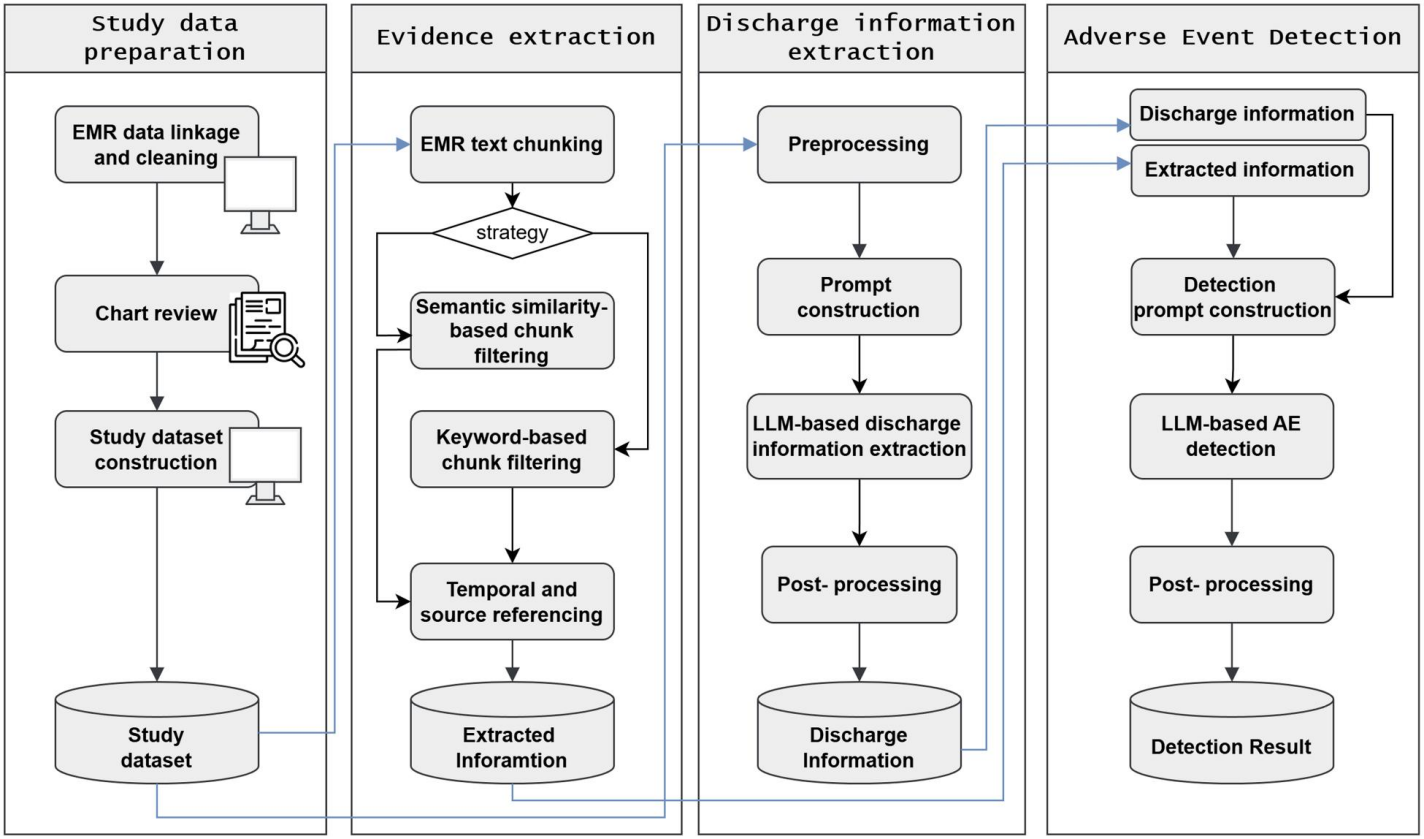
Framework for detecting hospital-acquired conditions using narrative EMR data. The framework consists of three modules: evidence extraction, discharge information extraction, and adverse event detection. Abbreviations used: AE, adverse event; EMR, electronic medical record; LLM, large language model.

Our proposed LLM-based PEAE detection framework consists of three modules, as illustrated in Figure 1.

#### Evidence extraction module

The evidence extraction module addresses a fundamental challenge in processing EMRs: converting extensive clinical narratives into concise, relevant text segments suitable for LLM analysis. This conversion is particularly crucial given that most LLMs have input token limitations,^27^ which necessitates careful selection and processing of clinical information. Our module processes every textual note during a patient’s hospital stay, encompassing up to one hundred twenty distinct hospital note types, including progress notes, nursing documentation, diagnostic reports, and consultation notes.

Our framework implements a two-step process for extracting relevant information. The first step is chunking, which divides the original raw clinical documents into smaller segments such as paragraphs, sentences, or word sequences. This segmentation enables more efficient processing for LLM analysis and helps maintain performance, which tends to degrade with increased input length.

The second step employs chunk selection strategies to reduce the amount of text used for prompt generation. We implemented and evaluated two distinct approaches. The first strategy uses keyword-based filtering, where we developed a filter based on our chart review guidelines that selects chunks containing PE-relevant keywords such as “PE,” “blood clot,” “thrombosis,” “pe,” “ctpe,” and their variants. This method ensures the capture of all explicitly mentioned relevant information.

Our second strategy employs semantic similarity-based filtering, which proves particularly valuable when specific keywords are absent but synonymous content is present. The fundamental assumption underlying this approach was that chunks containing information confirming PEAE presence should be semantically similar, whereas those carrying negating or unrelated information should be semantically distinct. This approach requires two components: example sentences that mimic content from various clinical note types (including nursing notes, assessment notes, discharge summaries, and diagnostic imaging reports) and a pre-trained language model that embeds both the chunked EMR text and example sentences into numerical vectors. The method calculates cosine similarity scores, between chunks and example sentences, ranking chunks and selecting the top *n* percent, where *n* is an adjustable threshold.

To optimize the embedding model for semantic filtering, we developed a triplet dataset inspired by the sentence-BERT approach.^28^ The dataset consists of three components designed to train the model to discern fine-grained distinctions between similar and dissimilar text chunks: anchors (text segments from EMR documents containing PE-related keywords), positive examples (chunks with similar PEAE-related keywords), and negative examples (either chunks from non-PE patient EMRs or negated versions of the anchors generated using Llama3).

We evaluated several embedding models, including BERT,^29^ BGE,^30^ BioClinicalBERT,^31^ E5,^32^ GTE,^33^ MPNet,^34^ and UAE.^35^ Our evaluation dataset comprised 9505 triplets, with 1811 reserved for evaluation and 7694 used for fine-tuning the best-performing model. This fine-tuning process improved the model’s adaptation to real-world data patterns.

#### Discharge information extraction module

The discharge information extraction module addresses the need to systematically process discharge summaries, which contain comprehensive information about each patients’ entire hospital stays, including details that might be closely related to PEAE. While these summaries provide valuable clinical information, their extensive nature poses challenges for efficient processing.

Our extraction module focused on six sections within discharge summaries. These were identified by analyzing our EMR data structure and known clinical practices.^36–39^ These components include *the reason for hospitalization*, *significant findings for the current admission*, *procedures and treatments provided*, *medical history*, *discharge condition*, and *patient and family instructions*. We employed a straightforward prompt instructing the LLM to parse the discharge summary to extract these details. The prompt was designed to return results in a structured JSON format.

#### PEAE detection module

Following the extraction process, our framework employs a detection module to determine whether the given EMR chart indicates the presence of PEAE. This module aggregates multiple data points extracted from previous modules, including evidence of PE, discharge information, and temporal information. These elements are stored in individual files to standardize input for further processing and are converted into structured prompts using a custom rule-based method implemented in Python. The method accommodates variations in prompt structure required by different LLMs. For example, Phi-3 employs “<|im_start|>“ and “<|im_end|>“ tags to delineate different parts of the prompt, while Mistral-7B uses “[INST]” tags for instructions (see Supplementary Materials “Prompt Engineering” section).

**Figure 2.**
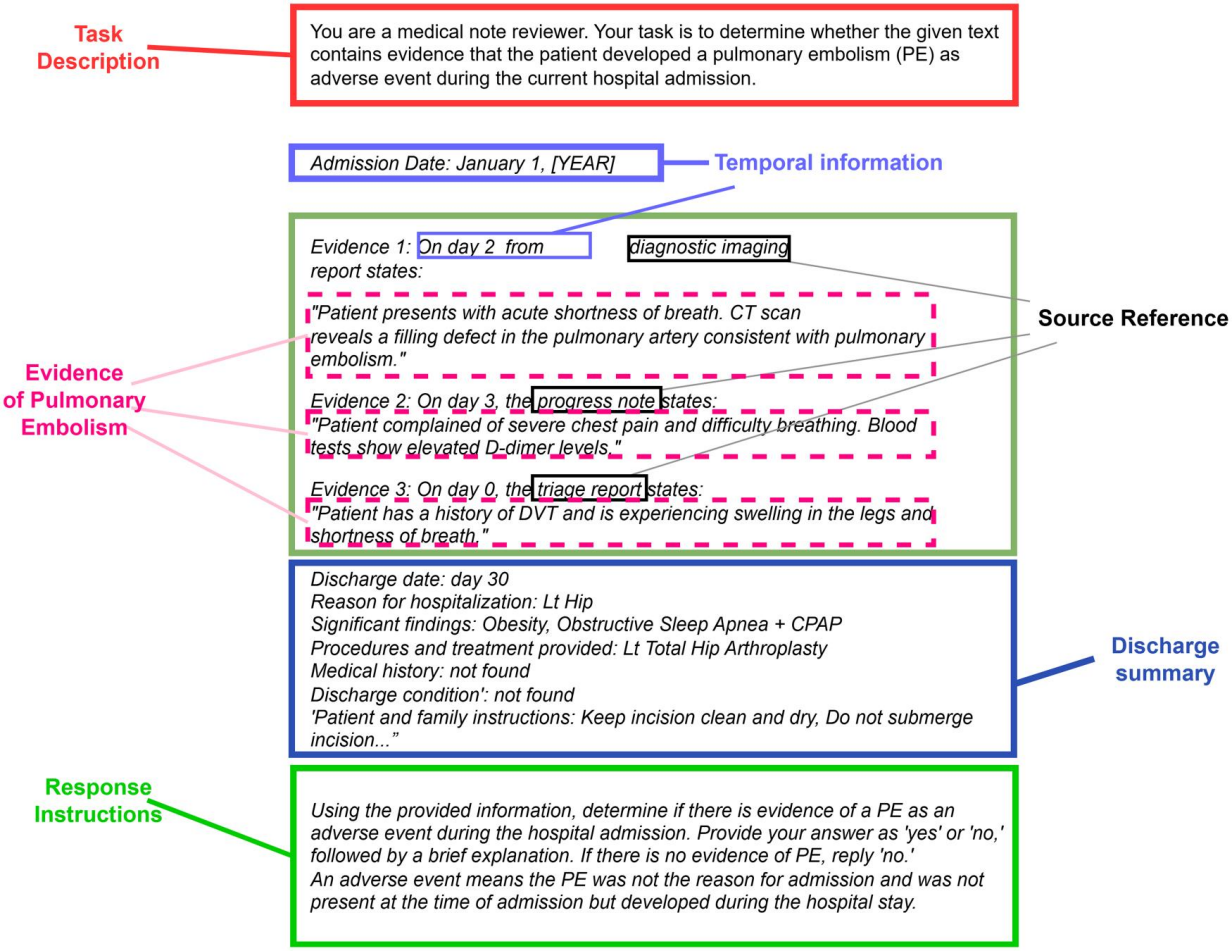
An example is prompt template for pulmonary embolism as an adverse event detection.

The PEAE detection module contains three main components: PEAE detection prompt construction, LLM inference, and post-processing classification. In the prompt construction phase, we transform the aggregated information fragments into structured prompts using a rule-based approach. Each prompt integrates information fragments according to specific LLM requirements (Figure 2). Then, we assessed four publicly available open-source LLMs for the PEAE identification task. These models include Llama3^17^ (Meta, Meta-Llama-3-8B-Instruct), Mistral 7B^18^ (Mistral AI, Mistral-7B-Instruct-v0.3), Gemma^19^ (Google, Gemma-7B-It), and Phi-3^20^ (Phi, Phi-3-Medium-128K-Instruct). Each model’s sequential response begins with a binary classification—”yes” or “no”—followed by a justification.

To derive a final classification, we apply a simple heuristic to the output: if the first sentence contains “yes,” we label the case as positive (1), and we otherwise label it as negative (0). In doing so, the detection module provides a comprehensive, automated assessment of each patient’s condition and the potential presence of PEAE.

### Experiment settings

All models for processing large-scale narrative EMRs were implemented within a secure computing environment using Python 3.11,^40^ PyTorch 2.3.0,^41^ and the Hugging Face Transformers 2.1 library.^42^

The experiments were conducted in a secure, health authority-approved computing environment with an NVIDIA H100 GPU cluster running CUDA 12.4. This infrastructure offered the necessary computational capacity to process and apply LLMs to the EMR data efficiently.

### Statistical analysis

We assessed the performance and validity of our proposed framework by comparing its results to those obtained through chart review. Evaluation metrics included positive predictive value (PPV), sensitivity, negative predictive value, specificity, and F1-score, each reported with a 95% confidence interval (CI). To compute these metrics, we compared the framework-generated PEAE outcome to the binary outcomes from the chart review. The 95% CI was estimated using 10 000 bootstrap resamples, with metrics calculated for each resample and summarized based on their distribution.

We further extended the analysis to assess the potential of our model for population-based surveillance of adverse events. Specifically, we evaluated the model’s ability to track PEAE incidence at different time granularities: year, year-quarter (Q), and year-month. For each granularity, we calculated the mean squared error, *R*^2^, and Pearson correlation coefficient to evaluate how well the predicted counts aligned with the actual counts.

## Results

### Characteristics of the datasets

Registered nurses conducted a comprehensive chart review of patient records (*n* = 10 066), identifying 40 cases of PEAE. Table 1 presents the demographic and clinical characteristics of the study population, comparing patients with confirmed PEAE cases to those without (non-PEAE). The analysis includes key variables such as age distribution, sex, death in hospital, number of comorbidities, and length of hospital stay. Statistical significance was assessed using appropriate tests, and *P-* values were reported for each comparison.

**Table 1.** The study cohort characteristics.

Variable	All (*n* = 10 066)	Confirmed PEAE (*n* = 40)	Confirmed non-PEAE (*n* = 10 026)	*P-*value
**Age (%)**				
<50	3022 (30.0)	11 (27.5)	3011 (30.0)	.931
50-64	2493 (24.8)	9 (22.5)	2484 (24.8)	
65-74	1921 (19.1)	9 (22.5)	1912 (19.1)	
≥75	2630 (26.1)	11 (27.5)	2619 (26.1)	
**Sex (%)**				
Female	5246 (52.1)	16 (40.0)	5230 (52.2)	.153
Male	4820 (47.9)	24 (60.0)	4796 (47.8)	
**Death in hospital (%)**	717 (7.1)	5 (12.5)	712 (7.1)	.204
**Number of comorbidities (%)**				
0	2416 (24.0)	6 (15.0)	2410 (24.0)	.352
1	2340 (23.2)	9 (22.5)	2331 (23.2)	
≥2	5310 (52.8)	25 (62.5)	5285 (52.7)	
**Length of stay (%)**				
1-4 days	4881 (48.5)	4 (10.0)	4877 (48.6)	<.001
≥5 days	5185 (51.5)	36 (90.0)	5149 (51.4)	

We evaluated two distinct filtering approaches to identify content relevant to PEAE. The first approach, a keyword-based filter, excluded chunks that lacked predefined PE-related keywords, reducing the total chunks across the dataset from 1 972 188 to 623 081 (Table 2). This filtering step decreased the median chunks per patient from 789 to 310.

**Table 2.** The word counts of different strategies.

Filtering step	Total chunks across the dataset	Median number of chunks per patient (IQR)	Median total word count per patient (IQR)
Raw text (before filter)	1 972 188	789.5 (428.8-1569.5)	17189.0 (9123.8-34822.0)
Keyword-based filtered	623 081	310.0 (195.0-505.0)	6854.5 (4189.75-11372.0)
Semantic filtered	22 749	9.0 (2.0-19.3)	216.5 (53.0-506.0)

The second approach applied a semantic similarity-based filter, utilizing cosine similarity algorithms to compare chunks against ten example sentences indicating PE conditions. The results of six different embedding models used in semantic filtering algorithms are presented in Supplementary Materials (see Table S1). The fine-tuned UAE embedding was used for all final evaluations. The reported triplet loss and accuracy values correspond to the validation set to ensure an unbiased assessment of the model’s performance. This semantic filtering method refined the dataset to 22 749 chunks, with a median of 9 chunks per patient and a median word count of 216 per patient. Both filtering strategies effectively reduced the dataset size while preserving clinically relevant PEAE information.

### PEAE detection results

We conducted a series of experiments to evaluate the performance of four LLMs under different conditions. These models were assessed based on two primary factors: (1) the inclusion or exclusion of discharge information and (2) keyword-based (KW) versus semantic similarity-based (SS) retrieval methods. Table 3 presents the performance metrics of each model, benchmarked against the manual chart review, which served as the gold standard. Additionally, we compared the LLM-based approach to a rule-based method using the 10th Edition of the International Classification of Diseases (ICD-10) codes as a secondary baseline.

**Table 3.** The PEAE detection performance from each model % (95% confidence interval).

Model	Retrieval	DCSM	PPV	Sensitivity	Specificity	NPV	F1 score
Gemma	KW	Yes	16.75 (11.85-21.92)	87.50 (76.32-97.14)	98.40 (98.16-98.63)	99.95 (99.91-99.99)	28.11 (20.91-35.48)
Gemma	KW	No	11.14 (7.87-14.53)	95.0 (86.96-100.00)	97.22 (96.9-97.52)	99.98 (99.95-100.0)	19.95 (14.53-25.41)
Gemma	SS	Yes	12.46 (8.86-16.16)	95.00 (86.96-100.00)	97.55 (97.25-97.83)	99.98 (99.95-100.0)	22.03 (16.28-27.83)
Gemma	SS	No	11.08 (7.83-14.49)	95.00 (87.18-100.0)	97.20 (96.88-97.5)	99.98 (99.95-100.0)	19.84 (14.55-25.13)
Llama3	KW	Yes	14.34 (10.04-18.88)	87.50 (76.19-97.22)	98.08 (97.81-98.34)	99.95 (99.91-99.99)	24.65 (18.12-31.27)
Llama3	KW	No	8.85 (6.33-11.57)	100.00 (100.0-100.0)	96.21 (95.86-96.56)	100.0 (100.0-100.0)	16.26 (11.84-20.75)
Llama3	SS	Yes	11.34 (8.12-14.88)	97.50 (91.49-100.0)	97.2 (96.88-97.51)	99.99 (99.97-100.0)	20.31 (15.05-25.77)
Llama3	SS	No	8.52 (6.07-11.31)	97.50 (91.67-100.0)	96.15 (95.78-96.5)	99.99 (99.97-100.0)	15.66 (11.32-19.97)
Mistral	KW	Yes	14.04 (10.08-18.21)	100.0 (100.0-100.0)	97.75 (97.46-98.02)	100.0 (100.0-100.0)	24.62 (18.36-30.94)
Mistral	KW	No	11.14 (7.97-14.55)	97.50 (91.67-100.0)	97.14 (96.83-97.45)	99.99 (99.97-100.0)	20.0 (14.73-25.29)
Mistral	SS	Yes	11.63 (8.31-15.15)	100.0 (100.0-100.0)	97.21 (96.9-97.52)	100.0 (100.0-100.0)	20.83 (15.38-26.25)
Mistral	SS	No	11.08 (7.9-14.44)	97.50 (91.67-100.0)	97.12 (96.8-97.43)	99.99 (99.97-100.0)	19.9 (14.58-25.13)
Phi3	KW	Yes	15.27 (9.38-21.54)	50.00 (34.21-65.71)	98.98 (98.79-99.16)	99.81 (99.73-99.89)	23.39 (14.81-31.85)
Phi3	KW	No	12.02 (7.47-16.95)	55.00 (39.47-70.59)	98.52 (98.29-98.74)	99.83 (99.75-99.91)	19.73 (12.77-26.72)
Phi3	SS	Yes	9.32 (6.68-12.11)	100.00 (100.0-100.0)	96.42 (96.08-96.77)	100.0 (100.0-100.0)	17.06 (12.47-21.65)
Phi3	SS	No	6.76 (4.83-8.88)	100.00 (100.0-100.0)	94.93 (94.5-95.33)	100.0 (100.0-100.0)	12.66 (9.16-16.18)
ICD-based method (Baseline)			37.5 (19.1-58.3)	14.52 (6.5-23.7)	99.85 (99.8-99.9)	99.48 (99.3-99.6)	20.93 (9.9-31.9)

This table summarizes the performance metrics of 4 models using different methods. The results were based on 10 000 bootstrap resamples to estimate the performance variability, with the metrics reported as the mean and 95% confidence intervals. The baseline method is a rule-based method based on ICD codes from a Discharge Abstract Database.

Abbreviations used: DCSM, inclusion of discharge information; ICD, International Classification of Diseases; KW: keyword-based retrieval methods; NPV, negative predictive value; PPV, positive predictive value; SS, semantic similarity-based retrieval methods.

Overall, none of the LLMs fully replicated the results of manual chart review, but they demonstrated varying levels of agreement with it across different retrieval conditions. Among the tested models, Gemma (keyword-based retrieval with discharge summaries) achieved the highest F1-score (28.11%), with a sensitivity of 87.50% and specificity of 98.40%. While this represents an improvement over other LLM configurations, the PPV remained relatively low across all models (ranging from 6.76% to 16.75%), suggesting that LLMs still generate a substantial number of false positives compared to manual abstraction.

The inclusion of discharge summaries generally improved F1 scores across models, likely due to the presence of more structured clinical narratives. For instance, for the Mistral model with KW retrieval, the F1 score increased from 20.00% (without discharge information) to 24.62% (with discharge information). Similarly, in Llama3, the F1 score improved from 16.26% to 24.65% when discharge summaries were included. Additionally, we compared the performance of KW retrieval with SS retrieval. The KW retrieval method generally outperformed SS retrieval across different models, particularly in F1 score and PPV. However, SS retrieval often achieved comparable or higher sensitivity, indicating that it captures more potential cases but at the cost of increased false positives.

Compared to the ICD-10-based method, LLMs achieved higher sensitivity (50% to 100%) but had lower PPV. The ICD-10-based approach had a higher PPV (37.5%) but much lower sensitivity (14.52%), missing many true cases. This tradeoff highlights the strengths and limitations of each method.

Table 4 summarizes the performance of the four LLMs in detecting adverse events using a yearly granularity, reflecting the models’ effectiveness in population-based surveillance and assessing how well they captured trends in PEAE incidence over time. We evaluated the models’ ability to track PEAE trends over time at different granularities. Table 4 summarizes the performance of each model, highlighting the highest correlations and lowest errors at the yearly granularity.

**Table 4.** Model performance for population-level surveillance at year granularity.

Model	Correlation	Mean squared error	*R* ^2^
Llama3	0.988	0.003	0.975
Phi3	0.978	0.005	0.953
Mistral	0.959	0.009	0.918
Gemma	0.921	0.019	0.828

## Discussion

The results of our method for PEAE detection using EMR notes demonstrated that all tested LLMs achieved high sensitivity and specificity, indicating a powerful ability to distinguish PEAE cases from non-PEAE cases. Additionally, their high sensitivity and NPV across all four models underscore their effectiveness as filtering tools, ensuring the retention of as many positive cases as possible while confidently excluding negative cases.

When comparing the KW chunking selection method with the semantic SS method, we found that the KW approach consistently achieved higher F1-scores and PPV. Our analysis showed that domain-specific keywords were highly effective in selecting relevant chunks of EMR notes for PEAE detection. For instance, KW chunk filtering achieved a higher F1 score (28.11% vs 22.03%) for the Gemma model compared with semantic similarity-based filtering. These results underscore the importance of incorporating domain knowledge into the chunk selection process for optimal performance.

We also observed that including the discharge information further enhanced the metrics, particularly the F1 score. It provided additional context that better captured relevant information and reduced misclassification.

The population-based analysis underscores the challenges of detecting trends in low-incidence-rate conditions like PEAE. While all LLM models demonstrated strong performance at the a yearly granularity, finer granularities such as monthly trends revealed greater variability in predictions. This variability is driven by the sparsity of PEAE cases in the dataset, which amplifies the impact of small fluctuations on model predictions. Larger aggregates at the yearly level reduce this variability, resulting in closer alignment between predicted and actual trends.

These findings suggest that the framework is well suited for identifying long-term trends in population-level surveillance, even in settings with imbalanced datasets. Future research could explore further refinements to enhance the framework’s performance for finer-grained analysis.

### Limitations

Almost all errors came from false positive cases. We further investigated the errors generated by our LLM-based PEAE detection framework and noticed several primary error types. These errors highlight the limitations faced by the proposed framework and tested LLMs in accurately interpreting complex medical information.

#### Contextual misinterpretation

The most common errors stemmed from the LLMs’ difficulty in accurately interpreting clinical context. This issue appeared in several ways. Temporal confusion led LLMs to misinterpret historical medical events as current conditions. Diagnostic uncertainty resulted in suspected conditions being frequently misclassified as confirmed diagnoses.

#### Limited capacity in compressing terminologies

We identified errors that reflected the framework’s medical knowledge and terminology limitations. Similar medical terms were occasionally conflated, such as misinterpreting pulmonary edema as PE. In addition, LLMs sometimes fail to interpret specialized sections of medical notes correctly. For example, in the “History of Present Illness” section, a past diagnosis of deep vein thrombosis was misinterpreted as evidence of current PE, despite contextual cues indicating successful treatment and no recent clot formation. Furthermore, certain treatments were overgeneralized as indicators of specific conditions, not accounting for their use in multiple medical contexts, such as interpreting the use of anticoagulants for PE prophylaxis as evidence of a confirmed diagnosis.

#### Near miss and implicit cases

The chart review involves reading the clinician’s notes to identify diagnoses and confirm the presence or absence of PEAE without making assumptions. Our chart reviewers determined PEAE using the information available in the chart. Unfortunately, sometimes, the chart may have insufficient information to determine whether a PEAE occurred. There were cases where patients received empiric therapy for suspected PE, but the diagnosis was not confirmed through documented follow-up diagnostics. Such cases were labeled as negative by the chart reviewers, as they relied solely on explicitly documented information and could not infer a diagnosis without confirmation explicitly noted in the chart.

#### Preprocessing and discharge information extraction

There are technical limitations in the preprocessing of medical data. Specifically, both keyword and semantic chunk search algorithms fail to preserve semantically consistent segments, leading to incorrect grouping of information. Additionally, the discharge information extraction module occasionally overfocused on primary diagnoses while omitting crucial secondary information, distorting the overall clinical picture.

#### Prompt engineering

The prompting strategies used in this study were designed to elicit binary outputs followed by brief explanations. While effective, future work could explore advanced techniques such as chain-of-thought prompting,^43^ which structures the explanation before the final response, potentially improving the interpretability and performance of the outputs. Additionally, few-shot prompting,^44^^,^^45^ may enhance the ability of LLMs to generalize across diverse scenarios. These adaptations could further optimize performance and robustness in detecting PEAE and other adverse events.

## Conclusion

Our proposed method demonstrates the strong potential of LLMs for detecting PEAE, with high sensitivity and specificity scores indicating effective discrimination between PEAE and non-PEAE cases across various classification thresholds. Among the 4 LLMs evaluated, Gemma achieved the highest F1 score, suggesting better overall balance between PPV and sensitivity in identifying PEAE cases from narrative EMR data. While all 4 models achieved high specificity and sensitivity, the low prevalence of PEAE contributed to a relatively high number of false positives, leading to a lower PPV. This suggests that these models cannot be relied upon solely to predict or select PEAE cases. Further chart review is necessary to confirm PEAE after initial LLM screening. Looking ahead, refining LLMs to enhance precision in detecting PEAE and other adverse events from EMRs will be essential as EMRs continue to be widely implemented worldwide.

## Supplementary Material

ocaf048_Supplementary_Data

## Data Availability

Owing to data-sharing policies of the data custodians, the dataset cannot be made publicly available. The complete code for this study is available from the corresponding author upon academically reasonable request.

## References

[1] Ford E , Carroll JA, Smith HE, Scott D, Cassell JA. Extracting information from the text of electronic medical records to improve case detection: a systematic review. J Am Med Inform Assoc. 2016;23:1007-1015. 10.1093/jamia/ocv180 26911811 PMC4997034

[2] Goldstein SL , Kirkendall E, Nguyen H, et al Electronic health record identification of nephrotoxin exposure and associated acute kidney injury. Pediatrics. 2013;132:e756-767-e767. 10.1542/peds.2013-0794 23940245

[3] Cheligeer C , Wu G, Lee S, et al BERT-based neural network for inpatient fall detection from electronic medical records: retrospective cohort study. JMIR Med Inform. 2024;12:e48995. 10.2196/48995 38289643 PMC10865188

[4] Zhao B , Zhang R, Chen D, et al A machine-learning-based approach for identifying diagnostic errors in electronic medical records. IEEE Trans Rel. 2024;73:1172-1186. 10.1109/Tr.2023.3330733

[5] Quan H , Khan N, Hemmelgarn BR, et al; Hypertension Outcome and Surveillance Team of the Canadian Hypertension Education Programs. Validation of a case definition to define hypertension using administrative data. Hypertension. 2009;54:1423-1428. 10.1161/Hypertensionaha.109.139279 19858407

[6] Xu Y , Lee S, Martin E, et al Enhancing ICD-code-based case definition for heart failure using electronic medical record data. J Card Fail. 2020;26:610-617. 10.1016/j.cardfail.2020.04.003 32304875

[7] Fleischmann-Struzek C , Thomas-Rüddel DO, Schettler A, et al Comparing the validity of different ICD coding abstraction strategies for sepsis case identification in German claims data. PLoS One. 2018;13:e0198847. 10.1371/journal.pone.0198847 30059504 PMC6066203

[8] Jolley RJ , Quan H, Jetté N, et al Validation and optimisation of an ICD-10-coded case definition for sepsis using administrative health data. BMJ Open. 2015;5:e009487. 10.1136/bmjopen-2015-009487 PMC4691777 26700284

[9] McBrien KA , Souri S, Symonds NE, et al Identification of validated case definitions for medical conditions used in primary care electronic medical record databases: a systematic review. J Am Med Inform Assoc. 2018;25:1567-1578. 10.1093/jamia/ocy094 30137498 PMC7646917

[10] Jette N , Reid AY, Quan H, Hill MD, Wiebe S. How accurate is ICD coding for epilepsy? Epilepsia. 2010;51:62-69. 10.1111/j.1528-1167.2009.02201.x 19682027

[11] Sheikhalishahi S , Miotto R, Dudley JT, et al Natural language processing of clinical notes on chronic diseases: systematic review. JMIR Med Inform. 2019;7:e12239. 10.2196/12239 31066697 PMC6528438

[12] Koleck TA , Dreisbach C, Bourne PE, Bakken S. Natural language processing of symptoms documented in free-text narratives of electronic health records: a systematic review. J Am Med Inform Assoc. 2019;26:364-379. 10.1093/jamia/ocy173 30726935 PMC6657282

[13] Sandhu N , Krusina A, Quan H, et al Automated extraction of weight, height, and obesity in electronic medical records are highly valid. Obes Sci Pract. 2024;10:e705. 10.1002/osp4.705 38263997 PMC10804327

[14] Tapson VF. Acute pulmonary embolism. N Engl J Med. 2008;358:1037-1052. 10.1056/NEJMra072753 18322285

[15] Thrombosis Canada. *Pulmonary Embolism: Diagnosis and Management*. Thrombosis Canada; 2013. https://thrombosiscanada.ca/guides/pdfs/PE.pdf

[16] Melton GB , Hripcsak G. Automated detection of adverse events using natural language processing of discharge summaries. J Am Med Inform Assoc. 2005;12:448-457. 10.1197/jamia.M1794 15802475 PMC1174890

[17] Llama Team, AI @ Meta. The Llama 3 herd of models. *arXiv preprint arXiv:2407.21783*. 2024. https://arxiv.org/abs/2407.21783

[18] Jiang AQ , Sablayrolles A, Mensch A, et al Mistral 7B. *arXiv preprint arXiv:2310.06825*. 2023. https://arxiv.org/abs/2310.06825

[19] Mesnard T , Hardin C, Dadashi R, et al Gemma: Open models based on Gemini research and technology. *arXiv preprint arXiv:2403.08295*. 2024. https://arxiv.org/abs/2403.08295

[20] Abdin M , Aneja J, Awadalla H, et al. Phi-3 technical report: A highly capable language model locally on your phone. *arXiv preprint arXiv:2404.14219*. 2024. https://arxiv.org/abs/2404.14219

[21] Wu G , Eastwood C, Sapiro N, et al Achieving high inter-rater reliability in establishing data labels: a retrospective chart review study. BMJ Open Qual. 2024;13:e002722. 10.1136/bmjoq-2023-002722 PMC11029337 38631818

[22] Wu G , Eastwood C, Zeng Y, et al Developing EMR-based algorithms to Identify hospital adverse events for health system performance evaluation and improvement: study protocol. PLoS One. 2022;17:e0275250. 10.1371/journal.pone.0275250 36197944 PMC9534418

[23] Friedman SM , Provan D, Moore S, Hanneman K. Errors, near misses and adverse events in the emergency department: what can patients tell us? CJEM. 2008;10:421-427. 10.1017/s1481803500010484 18826729

[24] Heit JA , Melton LJ, Lohse CM, et al Incidence of venous thromboembolism in hospitalized patients vs community residents. Mayo Clin Proc. 2001;76:1102-1110. 10.4065/76.11.1102 11702898

[25] Gee E. The National VTE Exemplar Centres Network response to implementation of updated NICE guidance: venous thromboembolism in over 16s: reducing the risk of hospital-acquired deep vein thrombosis or pulmonary embolism (NG89). Br J Haematol. 2019;186:792-793. 10.1111/bjh.16010 31168834

[26] Li HL , Zhang H, Chan YC, Cheng SW. Prevalence and risk factors of hospital acquired venous thromboembolism. Phlebology. 2024:2683555241297566. 10.1177/02683555241297566 39499060

[27] Levy M , Jacoby A, Goldberg Y. 2024. Same task, more tokens: the impact of input length on the reasoning performance of large language models. In: Ku L-W, Martins A, Srikumar V, eds. *Proceedings of the 62nd Annual Meeting of the Association for Computational Linguistics (Volume 1: Long Papers)*, 2024 Aug, Bangkok, Thailand. Association for Computational Linguistics; 2024:15339-15353.

[28] Reimers N , Gurevych I. Sentence-BERT: sentence embeddings using Siamese BERT-networks. In: *Proceedings of the 2019 Conference on Empirical Methods in Natural Language Processing and the 9th International Joint Conference on Natural Language Processing (EMNLP-IJCNLP)*, 2019 Nov, Hong Kong, China. 2019:3982-3992.

[29] Devlin J , Chang M-W, Lee K, et al BERT: pre-training of deep bidirectional transformers for language understanding. In: Burstein J, Doran C, Solorio T, eds. *Proceedings of the 2019 Conference of the North American Chapter of the Association for Computational Linguistics: Human Language Technologies, Volume 1 (Long and Short Papers)*, 2019 Jun, Minneapolis, MN. Stroudsburg, PA: Association for Computational Linguistics; 2019:4171-4186.

[30] Luo K , Liu Z, Xiao S, et al Landmark embedding: a chunking-free embedding method for retrieval augmented long-context large language models. In: Ku L-W, Martins A, Srikumar V, eds. *Proceedings of the 62nd Annual Meeting of the Association for Computational Linguistics (Volume 1: Long Papers)*, 2024 Aug, Bangkok, Thailand. Stroudsburg (PA): Association for Computational Linguistics; 2024:3268-3281.

[31] Alsentzer E , Murphy J, Boag W, et al Publicly available clinical BERT embeddings. In: Rumshisky A, Roberts K, Bethard S, Naumann T, eds. *Proceedings of the 2nd Clinical Natural Language Processing Workshop*, 2019 Jun 7, Minneapolis, MN. Stroudsburg, PA: Association for Computational Linguistics; 2019:72-78.

[32] Wang L , Yang N, Huang X, et al Text embeddings by weakly-supervised contrastive pre-training. *arXiv preprint arXiv:2212.03533*. 2022. https://arxiv.org/abs/2212.03533

[33] Li Z , Zhang X, Zhang Y, et al Towards general text embeddings with multi-stage contrastive learning. *arXiv, arXiv:2308.03281*. 2023. https://arxiv.org/abs/2308.03281

[34] Song K , Tan X, Qin T, Lu J, Liu T-Y. Mpnet: masked and permuted pre-training for language understanding. Adv Neural Inform Process Syst. 2020;33:16857-16867.

[35] Li X , Li J. AnglE-optimized text embeddings. *arXiv preprint arXiv:2309.12871*. 2023. https://arxiv.org/abs/2309.12871

[36] Wimsett J , Harper A, Jones P. Review article: components of a good quality discharge summary: a systematic review. Emerg Med Australas. 2014;26:430-438. 10.1111/1742-6723.12285 25186466

[37] Kind AJH , Smith MA. Documentation of mandated discharge summary components in transitions from acute to subacute care. In: Henriksen K, Battles JB, Keyes MA, Grady ML, eds. *Advances in patient safety: new directions and alternative approaches*. Vol. 2, Culture and Redesign. Rockville, MD: Agency for Healthcare Research and Quality; 2008:188-197. 21249900

[38] Dean SM , Gilmore-Bykovskyi A, Buchanan J, Ehlenfeldt B, Kind AJ. Design and hospital wide implementation of a standardized discharge summary in an electronic health record. Jt Comm J Qual Patient Saf. 2016;42:555-561. 10.1016/S1553-7250(16)30107-6 28334559 PMC5367268

[39] Weetman K , Spencer R, Dale J, Scott E, Schnurr S. What makes a “successful” or “unsuccessful” discharge letter? Hospital clinician and General Practitioner assessments of the quality of discharge letters. BMC Health Serv Res. 2021;21:349. 10.1186/s12913-021-06345-z 33858383 PMC8048210

[40] Python Software Foundation. *Python 3.11.0 [Internet]*. Wilmington, DE: Python Software Foundation; 2022. https://www.python.org/downloads/release/python-3110/

[41] Paszke A , Gross S, Massa F, et al PyTorch: an imperative style, high-performance deep learning library. In: Wallach H, Larochelle H, Beygelzimer A, d'Alché-Buc F, Fox E, Garnett R, eds.*Advances in Neural Information Processing Systems 32 (NeurIPS 2019)*. Curran Associates, Inc.; 2019:8024-8035.

[42] Wolf T , Debut L, Sanh V, et al *Proceedings of the 2020 Conference on Empirical Methods in Natural Language Processing: System Demonstrations*, 2020 Oct, Online. Stroudsburg, PA: Association for Computational Linguistics; 2020:38-45. https://aclanthology.org/2020.emnlp-demos.6/

[43] Wei J , Wang X, Schuurmans D, et al Chain of Thought Prompting Elicits Reasoning in Large Language Models. In: Koyejo S, Mohamed S, Agarwal A, Belgrave D, Cho K, Oh A, eds. *Advances in Neural Information Processing Systems 35 (NeurIPS 2022),* 2022 Dec, New Orleans, LA. Curran Associates, Inc.; 2022:24824-24837.

[44] Agrawal M , Hegselmann S, Lang H, Kim Y, Sontag D. Large language models are few-shot clinical information extractors. In: *Proceedings of the 2022 Conference on Empirical Methods in Natural Language Processing*, 2022 Dec, Abu Dhabi, United Arab Emirates. Stroudsburg, PA: Association for Computational Linguistics; 2022:1998-2022.

[45] Brown T , Mann B, Ryder N et al Language models are few-shot learners. In: Larochelle H, Ranzato M, Hadsell R, Balcan MF, Lin H, eds. *Advances in Neural Information Processing Systems 33 (NeurIPS 2020)*, 2020 Dec, Vancouver, Canada. Curran Associates, Inc.; 2020:1877-1901.

