# Supplementary material for: Utilizing large language models for detecting hospital-acquired conditions: an empirical study on pulmonary embolism
Source: J Am Med Inform Assoc. 2025 Mar 19;32(5):876–84. doi: 10.1093/jamia/ocaf048 (PMC12012340; doi:10.1093/jamia/ocaf048)
Supplement: ocaf048_Supplementary_Data [file ocaf048_supplementary_data.docx]

# Supplementary Document

## Model Selection for EMR Document Retrieval Task

We experimented to evaluate various embedding models for retrieving relevant text chunks from Electronic Medical Records (EMRs) that contain information about Pulmonary Embolism (PE). The evaluation was based on two key metrics: triplet loss and accuracy.

In this task, we used text chunks from EMR documents that contained PE-related keywords as the anchor. Positive examples were chunks that also included similar PE-related keywords, while negative examples were either chunks from EMRs of non-PE patients or negated versions of the anchor, which were generated using a large language model (LLM). For instance, "The patient has PE" was transformed into "The patient does not have PE." The experiment was based on two key assumptions. First, chunks that confirm the presence of PE should be semantically like one another. Second, chunks that either negate PE or are unrelated to it should be semantically different from those confirming PE.

We used an automatically generated dataset, created according to these assumptions without human validation, to evaluate model performance. This dataset, comprising over 1,000 triplets of anchor, positive, and negative examples, was designed to reflect real-world data patterns. The training and validation procedure follows an 80:20 train-test split ratio, and we additionally take 10% of training set as development set to monitor the model training procedure.

To assess the models, we measured triplet loss and accuracy. Triplet loss evaluates the model's ability to distinguish between positive and negative examples of the anchor, with a lower triplet loss indicating better performance. Accuracy, however, reflects how well the model classifies the relationships between anchor-positive and anchor-negative pairs, with higher accuracy representing better model performance.

We conducted model evaluation on validation set and the result as described in Table S.1.

By leveraging this automatically generated dataset, we aimed to identify the most effective embedding model for retrieving PE-related information from EMR documents.

Table S. 1. Embedding Model Evaluation on validation set.

| **Embedding Model Name** | **Triplet loss** | **Accuracy** |
| --- | --- | --- |
| bert_base | 0.68 | 0.70 |
| bge_v15 | 0.87 | 0.90 |
| bio_clinicalbert | 0.51 | 0.81 |
| e5_base_v2 | 0.93 | 0.88 |
| gte_base | 0.92 | 0.88 |
| mpnet_base | 0.89 | 0.77 |
| uae | 0.27 | 0.90 |
| uae_finetuned | 0.02 | 0.99 |

## Chunk selection

The chunk selection algorithm is designed to identify the most relevant chunks of text by calculating their cosine similarity to a set of anchor examples. First, we compute the cosine similarity (equation 1) between the embeddings of each chunk $\boldsymbol{M}_{\boldsymbol{1}}$ and 10 predefined anchor examples $\boldsymbol{M}_{\boldsymbol{2}}$, resulting in a similarity matrix $\boldsymbol{S}$ where each row represents a chunk, and each column corresponds to one anchor. Next, we apply mean pooling (algorithm 1, step 1) across the columns (anchor examples) for each row, yielding an average similarity score for each chunk. This score reflects how similar a chunk is, on average, to the given anchor examples. This results a vector $\boldsymbol{\mu}$, where each row contains the average similarity score of the corresponding chunk. Finally, we rank the chunks based on these scores and select the top-ranked chunks for each patient, focusing on the most relevant chunks based on a predefined threshold $\boldsymbol{p}$.

$$\begin{aligned} S=\frac{M_{1}M_{2}^{\top}}{|{|M}_{1}\left| |\cdot|{|M}_{2} \right||}\#\left( 1 \right) \end{aligned}$$

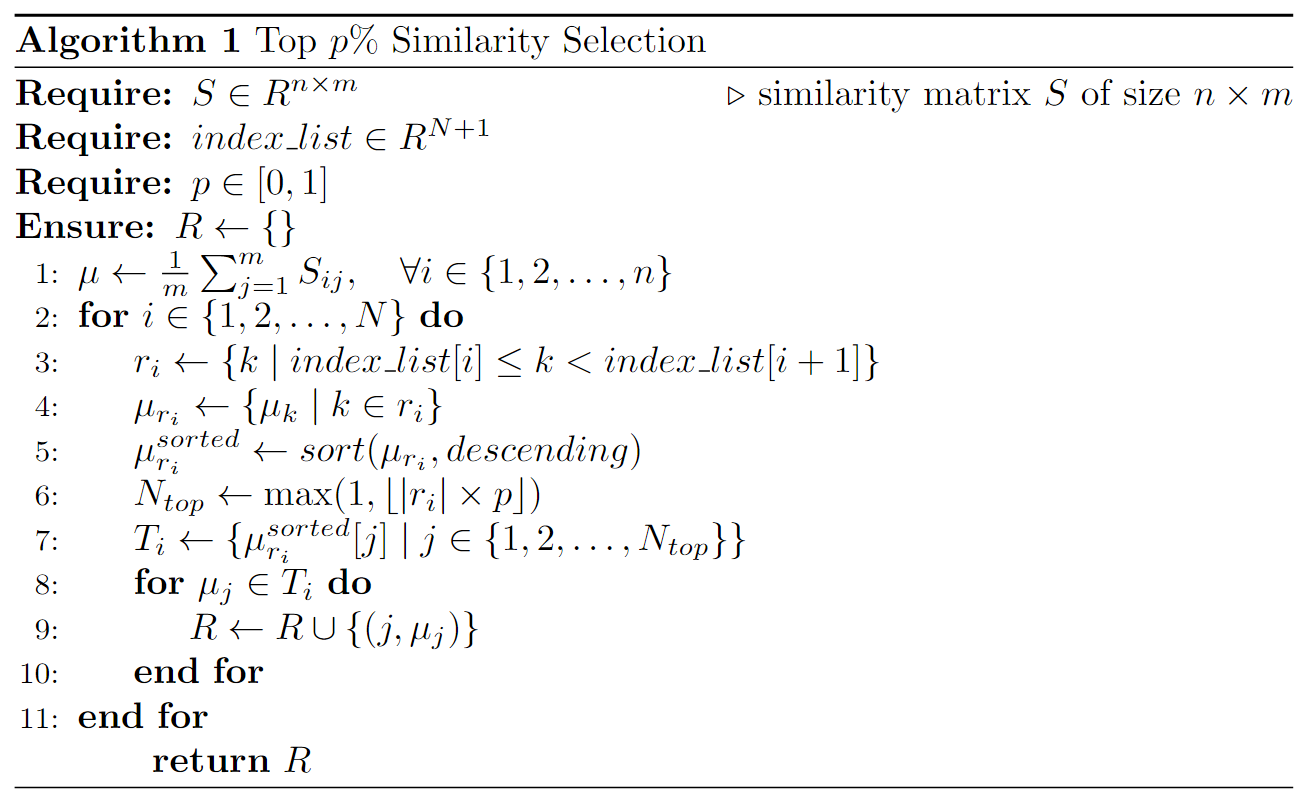


Algorithm 1. Top p% similar chunk selection.


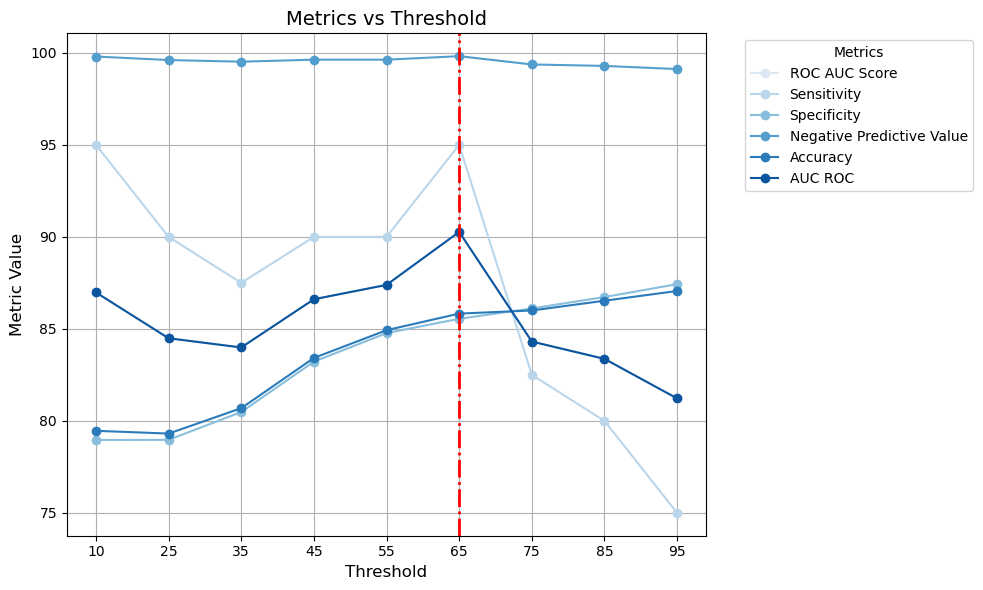


Figure S. 1. The impact of threshold on different performance metrics.

We observe that constructing a prompt without employing any chunking or chunk selection method leads to exceeding the token limits. From Figure S. 1, we can see that while we set the semantic similarity threshold at 65%, our framework would have better performance on different metrics in general.

We manually developed ten examples (Table S.2.) for our chunk selection algorithms, and our team thoroughly reviewed each one.

Table S. 2. Example anchors used in this study.

| **ID** | **Description** |
| --- | --- |
| 1 | *A CT scan confirmed a small burden of pulmonary embolism in the lower right lobe.* |
| 2 | *Radiological findings showed a subsegmental pulmonary embolism during a routine scan.* |
| 3 | *The patient was diagnosed with an acute pulmonary embolism.* |
| 4 | *Imaging revealed a pulmonary embolism with a small clot burden in the pulmonary arteries.* |
| 5 | *He was found to have a pulmonary embolism during his medical evaluation.* |
| 6 | *A filling defect indicative of a pulmonary embolism was observed in the segmental arteries.* |
| 7 | *The imaging report noted a mild burden of subsegmental PE in the posterior basal arteries.* |
| 8 | *A pulmonary embolism with a small clot burden was identified in the patient’s right lung.* |
| 9 | *Radiology results indicated a positive PE involving the central and segmental arteries.* |
| 10 | *A positive diagnosis of pulmonary embolism was made following the detection of clots in the segmental arteries.* |

## Hyperparameters

We studied the impact of hyperparameters on LLMs, and here we demonstrate the result from Llama3.

**Temperature:** This parameter controls the randomness of the predictions. Lower values (closer to 0) make the model more deterministic, while higher values (closer to 1) allow for more randomness in the output.


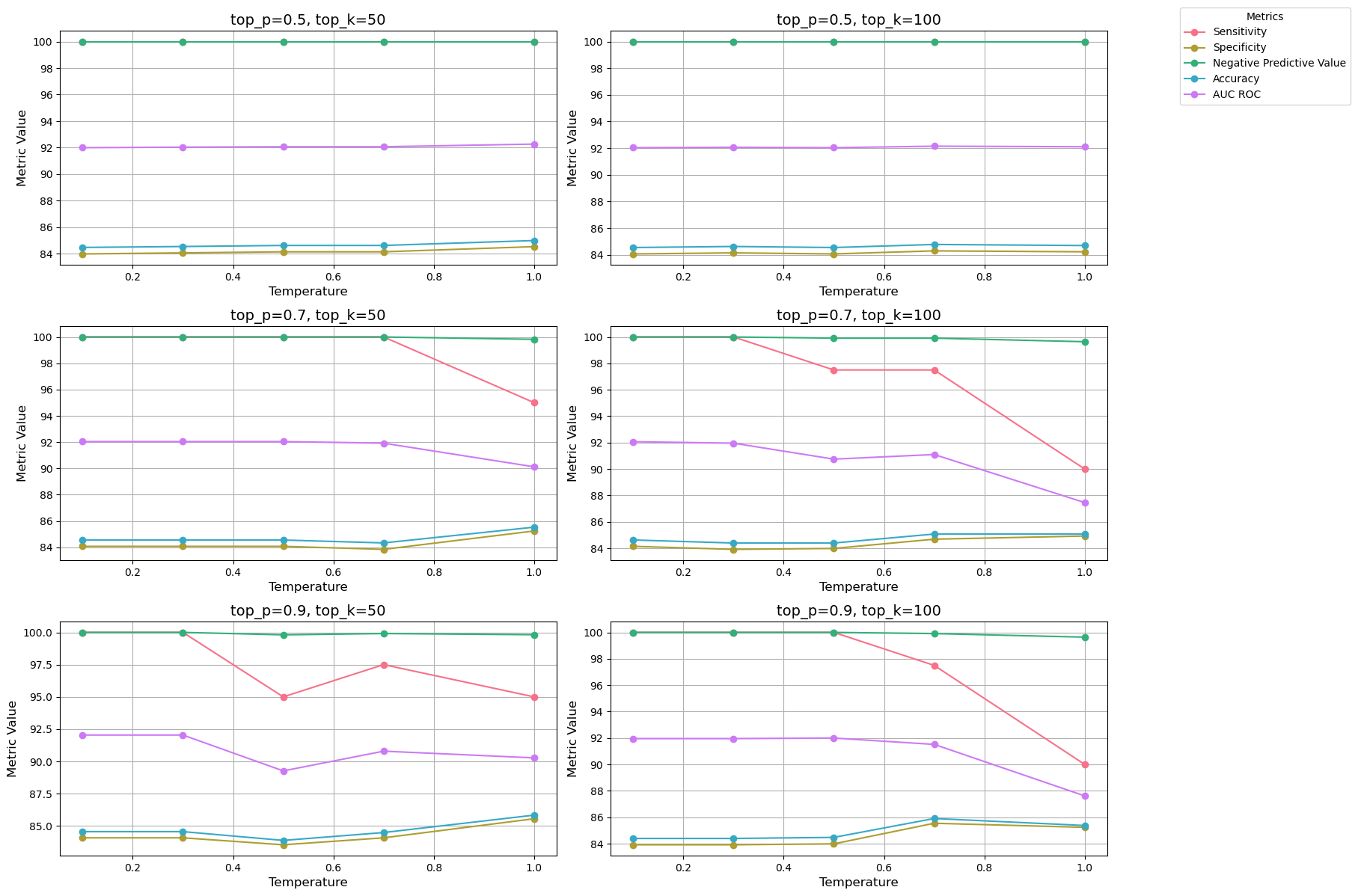


Figure S. 2. Impact of top-p, top-k, and temperature on performance metrics.

**Top-p (Nucleus Sampling):** This parameter controls the cumulative probability for selecting the next token in a sequence. For example, when set to 50%, the model selects tokens from the smallest set of options whose combined probability reaches 50%. Increasing this value to 70% or 90% allows for more variability by expanding the range of token probabilities considered.

**Top-k:** This parameter limits the number of token options the model considers for each prediction. For instance, a value of 50 means the model will only consider the 50 most likely next tokens, while increasing it to 100 expands that range to the top 100 tokens.

## Predicted PE trend and actual PE trend over time

Although our framework generated many false positives, after manually reviewing these errors, we believe our framework can be extended to population-based surveillance by providing relative data rather than an absolute value.

In this study, we evaluated the ability of several predictive models to track adverse events (specifically pulmonary embolism, PEAE) at the population level using a dataset of 10066 patients, where only 40 cases of PEAE were identified. Despite the significant imbalance in the data, our models demonstrated high sensitivity, identifying the majority of PEAE cases without too many misses. However, the PPV remained low, a common outcome in low-prevalence settings due to the high number of false positives. To determine whether these models could be used for population-level surveillance, we compared the predicted vs actual PEAE incidence stratified by year, year-quarter (Q), and year-month.

To compare the model predictions with the actual PEAE counts identified through manual chart review, we first normalized the PEAE counts. The normalization was performed using min-max scaling, where both the actual and predicted PEAE counts were transformed to a range of 0 to 1. This normalization allowed us to directly compare the distributions of the actual and predicted values at different granularities, regardless of their original scales.

The MSE was used to quantify the average squared difference between the actual and predicted values, providing a measure of prediction error. R-squared (R²) quantified the proportion of variance in the actual PEAE counts explained by the model, indicating how well the model captures the variability of the data. Pearson correlation was used to assess the linear relationship between the predicted and actual PEAE counts, highlighting how closely the model's predictions follow the actual trend.

The results were visualized using kernel density estimation (KDE) plots to compare the distribution of actual vs. predicted PEAE counts for each time granularity. Separate KDE plots were generated for year, year-quarter (Q), and year-month granularities. To ensure consistency, the same color scheme was used across all plots for each predictive model, and a single legend was positioned in the top-right corner for clear reference. This visualization allowed us to observe how closely the models' predicted distributions aligned with the actual distribution across different time granularities.

Table S. 3. Performance of LLMs in detecting PEAE trends over time, showing configurations with the highest correlation and lowest error metrics for each model. KW: keyword-based retrieval; SS: semantic similarity-based retrieval; DCSM: with discharge summary; NO_DCSM: without discharge summary. LLA3: Meta-Llama-3-8B-Instruct; PHI3: Phi-3-Medium-128K-Instruct; MISTRAL: Mistral-7B-Instruct-v0.3; GEMMA: Gemma-7B-It.

| **Experiment** | **KW_DCSM_GEMMA** | **KW_W/O_DCSM_GEMMA** | **SS_DCSM_GEMMA** | **SS_W/O_DCSM_GEMMA** | **KW_DCSM_LLA3** | **KW_W/O_DCSM_LLA3** | **SS_DCSM_LLA3** | **SS_W/O_DCSM_LLA3** | **KW_DCSM_MISTRAL** | **KW_W/O_DCSM_MISTRAL** | **SS_DCSM_MISTRAL** | **SS_W/O_DCSM_MISTRAL** | **KW_DCSM_PHI3** | **KW_W/O_DCSM_PHI3** | **SS_DCSM_PHI3** | **SS_W/O_DCSM_PHI3** |
| --- | --- | --- | --- | --- | --- | --- | --- | --- | --- | --- | --- | --- | --- | --- | --- | --- |
| **Correlation (Year)** | 0.909 | 0.921 | 0.9 | 0.923 | 0.988 | 0.958 | 0.922 | 0.965 | 0.944 | 0.958 | 0.946 | 0.959 | 0.671 | 0.813 | 0.963 | 0.978 |
| **Correlation (Year-Month)** | 0.527 | 0.462 | 0.467 | 0.469 | 0.629 | 0.421 | 0.408 | 0.431 | 0.565 | 0.467 | 0.471 | 0.461 | 0.331 | 0.107 | 0.525 | 0.419 |
| **Correlation (Year-Q)** | 0.564 | 0.558 | 0.59 | 0.569 | 0.724 | 0.595 | 0.53 | 0.626 | 0.695 | 0.598 | 0.587 | 0.587 | 0.335 | 0.472 | 0.562 | 0.453 |
| **MSE (Year)** | 0.024 | 0.019 | 0.025 | 0.02 | 0.003 | 0.01 | 0.017 | 0.008 | 0.012 | 0.009 | 0.012 | 0.009 | 0.071 | 0.059 | 0.008 | 0.005 |
| **MSE (Year-Month)** | 0.082 | 0.094 | 0.083 | 0.095 | 0.062 | 0.087 | 0.103 | 0.096 | 0.103 | 0.108 | 0.122 | 0.11 | 0.15 | 0.097 | 0.114 | 0.098 |
| **MSE (Year-Q)** | 0.131 | 0.105 | 0.107 | 0.097 | 0.103 | 0.074 | 0.079 | 0.069 | 0.076 | 0.082 | 0.084 | 0.086 | 0.159 | 0.103 | 0.086 | 0.097 |
| **R-Squared (Year)** | 0.787 | 0.828 | 0.774 | 0.817 | 0.975 | 0.909 | 0.849 | 0.926 | 0.891 | 0.916 | 0.894 | 0.918 | 0.362 | 0.464 | 0.926 | 0.953 |
| **R-Squared (Year-Month)** | -1.061 | -1.375 | -1.1 | -1.386 | -0.556 | -1.179 | -1.587 | -1.42 | -1.603 | -1.728 | -2.058 | -1.771 | -2.784 | -1.447 | -1.856 | -1.471 |
| **R-Squared (Year-Q)** | -0.827 | -0.457 | -0.498 | -0.348 | -0.429 | -0.034 | -0.102 | 0.036 | -0.057 | -0.15 | -0.176 | -0.194 | -1.217 | -0.436 | -0.199 | -0.354 |

By conducting this multi-granularity analysis, we aimed to evaluate the model's potential for population-level surveillance. While the model showed strong performance at the year granularity, with predicted distributions closely aligning with actual values, its accuracy decreased at finer granularities, such as year-quarter and year-month. This decline in performance suggests that the model may capture long-term trends more effectively than short-term fluctuations. The calculated metrics (MSE, R², and Pearson correlation) further supported this observation, with higher correlations and lower errors at the year level, and more variability in predictions at the monthly level.


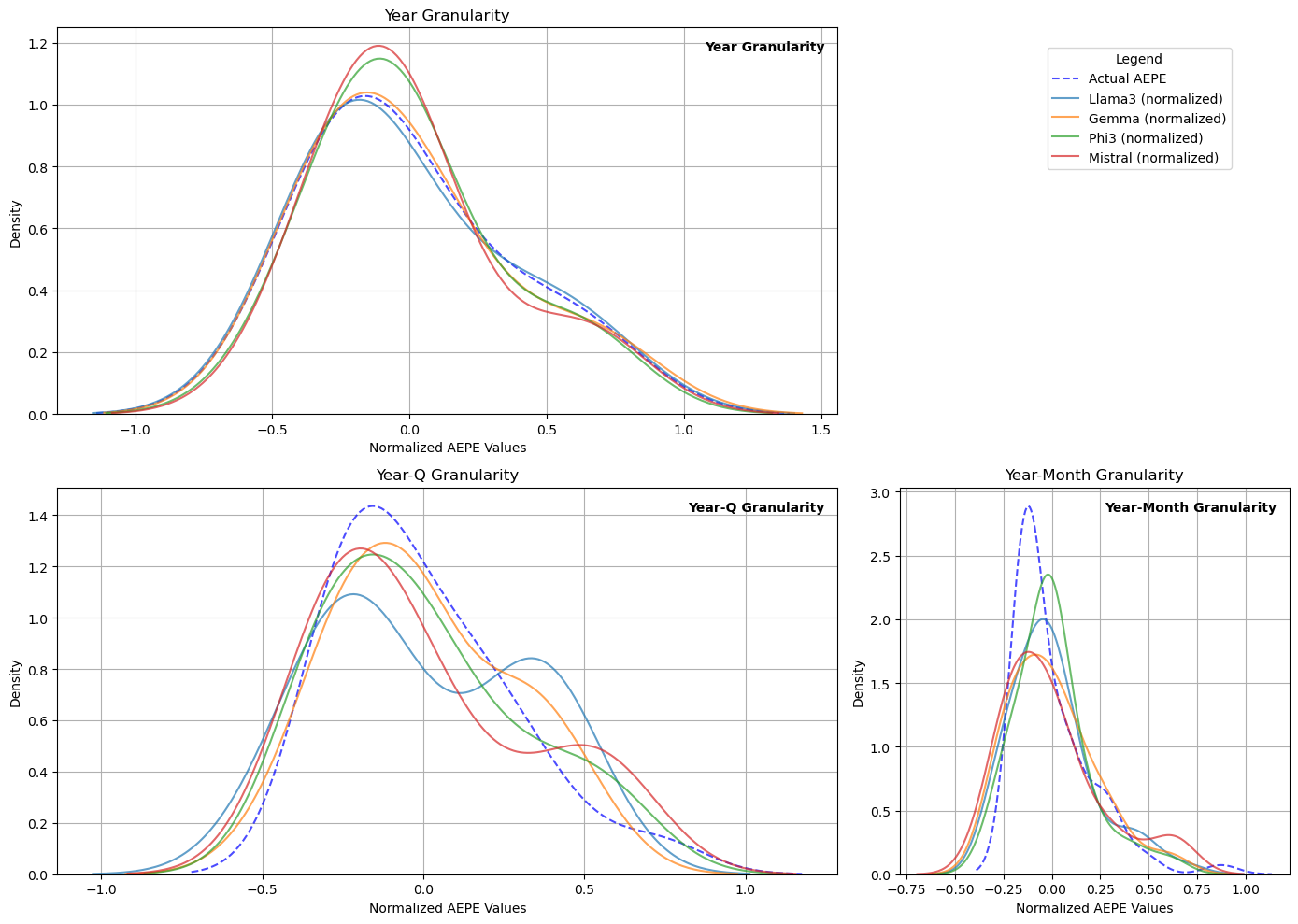


Figure S. 3. Density plots that compare the actual vs. predicted PEAE values for LLMs across three different time granularities—year, year-quarter (Q), and year-month.

However, when we examined the models at finer time granularities, such as year-quarter and year-month, performance dropped significantly. Correlation values were lower, and negative R-squared values were observed in many cases, indicating that the models struggled to predict short-term trends accurately. The poor performance at these finer granularities suggests that the models may not be able to capture sporadic fluctuations in PEAE incidence, which are more pronounced at the monthly or quarterly levels. These results highlight the strength of the models in year-level surveillance but also point to their limitations when predicting PEAE trends at more granular time scales. While they can be trusted for annual incidence monitoring, improving the models for quarterly or monthly predictions may require further refinement, such as incorporating additional features, applying time-series models, or reducing the impact of data imbalance. In conclusion, while the models show great promise for annual population-level surveillance of AEPE, their utility at the monthly and quarterly levels is limited. Future efforts should focus on improving precision and capturing more granular trends to better support real-time public health surveillance.
